# Rapid Characterization of Local Shape Memory Properties through Indentation

**DOI:** 10.1038/s41598-017-13434-9

**Published:** 2017-11-01

**Authors:** Peizhen Li, Haluk E. Karaca, Yang-Tse Cheng

**Affiliations:** 1 Department of Mechanical Engineering, University of Kentucky, Lexington, Kentucky 40506-0503 USA; 2 Department of Chemical and Materials Engineering, University of Kentucky, Lexington, Kentucky 40506-0046 USA

## Abstract

Shape memory alloys (SMAs) have the ability to show large recoverable shape changes upon temperature, stress or magnetic field cycling. Their shape memory, material and magnetic properties (e.g. transformation temperatures, strain, saturation magnetization and strength) determine their prospects for applications from small-scale microelectromechanical systems to large scale aerospace and biomedical systems. It should be noted that properties of SMAs are highly temperature dependent. Generally, the conventional mechanical characterization methods (e.g, tension, compression, and torsion) are used on bulk samples of SMAs to determine those properties. In this article, it will be shown that indentation technique can be used as an alternative rapid method to determine some of the important shape memory properties of SMAs. Indentation response of a high-temperature NiTiHf alloy was determined as a function of temperature. A clear relationship between the work recoverable ratio and transformation temperatures, superelastic and plastic behavior was observed. This work shows that indentation response can be used to measure local superelasticity response, determine phase transformation temperatures and reveal the temperature intervals of the deformation mechanisms of shape memory alloys.

## Introduction

Shape memory alloys (SMAs) are well known for their unique properties such as shape memory effect (SME) and superelasticity (SE), whereby large deformations can recover through reversible phase transformations. While there are many SMAs, the most commonly studied SMAs over the past 50 years are NiTi based alloys that have a relatively large reversible strain (~8%). NiTi alloys have been exploited for applications in several fields, including biomedical devices^1–3^, civil engineering structures^4,5^, and microelectromechanical systems (MEMS)^6,7^. Since the design of SMA-based MEMS and elaborate medical devices requires the characterization of material performance on small-scales, several tools that can quickly and accurately probe the mechanical properties on small-scale materials have been developed^8^. A powerful method of probing micro- and nano-scale mechanical properties of materials is the instrumented indentation which measures local mechanical properties, such as Young’s modulus, hardness, and strain hardening coefficient^9^.

Previous nanoindentation studies on SMAs were mostly conducted at room temperature to determine indentation hardness, depth recovery ratio and superelasticity^8,10–12^. It was found that in superelastic SMAs, the indentation depth imposed by spherical indenters can be almost fully recovered upon heating, while deformation induced by Berkovich indenter can only be partially recovered^10^. Moreover, the instrumented indentation method was also used to demonstrate indentation induced two-way shape memory effect^13,14^ and determine phase transformations^15^. Recent advances in high temperature indentation instrumentation have stimulated a broad interest in the indentation response of materials over a wide temperature range. In 1995, Poisl *et al*. performed elevated-temperature nanoindentation by placing a commercial nanoindenter in a temperature-controlled room which could be heated from room temperature to 34 °C^16^. Later, Suzuki and Ohmura built an optimized high temperature (up to ~600 °C) indentation instrument with a fast heating rate which affects the sensitivity during testing^17^. Later, commercial indentation equipment was developed to overcome sample oxidation and system thermal drift, allowing both nano and micro-indentation measurements up to 750 °C.

High temperature nanoindentation measurements have been conducted on many alloys such as fused silica^18–20^, aluminum^19^, Titanium^18^, copper^19^, and Ni-based super alloys^21^. A number of researchers also studied NiTi at elevated-temperatures^22–26^. The remnant indent depth ratios (depth after unloading/depth at peak load) were measured as a function of temperature for NiTi alloys under a spherical indenter, and it was found that the relative low remnant indent depth ratio is indicative to the occurrence of superelasticity^22^. It was confirmed that this procedure could also be applied to evaluate thin films of binary and ternary NiTi-based (Hf, Cu) shape memory alloys over a range of temperature (up to 400 °C)^23^. The indentation-induced SE effect was also characterized under Berkovich indenter at elevated temperature by Zhang *et al*.^25^, and they observed that the microscopic superelastic behavior of NiTi thin-film occurred at about 100 K above the austenite transformation finish temperature (A_f_) which was due to the high hydrostatic pressure under the indenter. Recently, it was found that the phase transformation temperatures and other characteristics of shape memory alloy samples could be estimated by utilizing the instrumented indentation techniques as well as *in situ* nano-compression in a transmission electron microscope (TEM)^27,28^. In addition, stress-induced phase transformations on shape memory alloys have also been studied at the nano-scale by using multiple nanoindentation techniques^29^. These recent studies clearly show that instrumented indentation techniques can be used to characterize the mechanical response of bulk and thin film NiTi alloys. In this study, Ni_50.3_Ti_29.7_Hf_20_ alloy was selected since it has higher strength than other NiTi-based alloys and shows superelasticity at high temperatures of about 200 °C^30–33^. The main aim of this study is to characterize the mechanical behavior of NiTiHf alloys as a function of temperature under a spherical indenter in micro-scale and compare that with compression experiments at macro-scale.

## Experimental Results

### Compression Stress-Strain and Phase Diagram

The start and finish temperatures of martensite and austenite of the aged NiTiHf alloy are measured as 186, 171, 195, and 205 °C (M_s_, M_f_, A_s_, A_f_), respectively from the DSC results. A typical SMA exhibits phase transformation between martensite and austenite phases, depending on applied stress and temperature. There are typically five stages of deformation: I) (T < M_f_) martensite reorientation (MR), no recovery; II) (M_f_ < T < A_f_) phase transformation (PT) behavior or variant reorientation (VR) with no or partial recovery; III) (A_f_ < T < T_SE_) superelastic (SE) behavior with full recovery; IV) (T_SE_ < T < M_d_) phase transformation with plastic deformation (PD) results in partial recovery or no recovery; V) (M_d_ < T) plastic deformation of austenite with no recovery. Here, the M_d_ temperature is defined as the maximum temperature at which SMAs can no longer transform to martensite. It should be noted that in all stages, the recovery amount will depend on the level of maximum loading strain/stress. The stages above were determined by assuming that the loading is only continued till the end of phase transformation.

**Figure 1:**
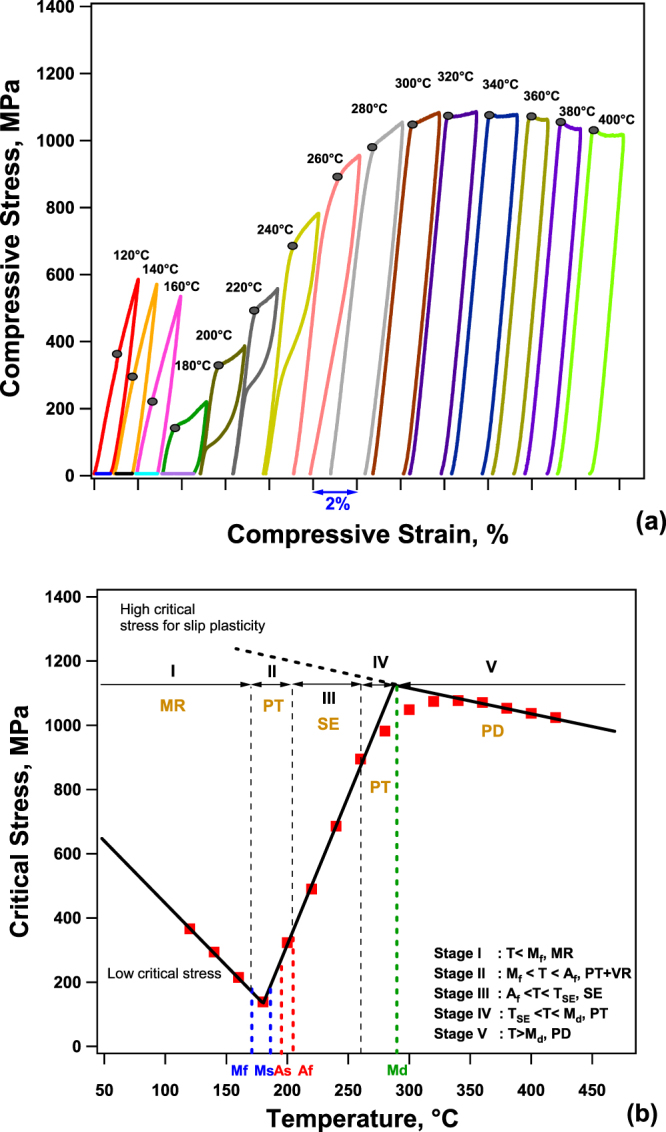
Phase transformation diagram of aged Ni_50.3_Ti_29.7_Hf_20_. The stress-strain curves showing temperature dependency of the critical stress (**a**); relationship of critical stress and transformation temperatures (**b**).

The compressive response of NiTiHf as a function of temperature is shown in Fig. 1a and a detailed phase transformation diagram of bulk NiTiHf alloy is presented in Fig. 1b, where critical stresses for martensite reorientation, martensitic transformation or slip are shown as a function of temperature. Critical stresses, ${\sigma }_{c}^{T}$, are extracted from Fig. 1a by using the tangent method. It should be noted that one sample was tested from 120 to 400 °C with temperature intervals of 20 °C. At 120 °C, which is below M_f_ of 171 °C, no strain recovery was observed upon unloading. However, SME strain was attained once heating the sample above the A_f_. As shown in the Fig. 1a, the critical stress for the martensite reorientation decreases with increasing temperature from 120 to 180 °C, presumably due to the increased mobility of internal twins and martensite plate boundaries. Only partial/no recovery was observed upon unloading below M_s_ and deformation can be fully recovered while heating above A_f_. At the temperature close to M_s_ of 186 °C, the stress-strain curve after the initial linear elastic deformation starts to exhibit a plateau like response at 180 °C which can be attributed to the initial stage of stress-induced martensite (SIM) being formed during loading, and followed by a small amount of elastic recoverable strain upon unloading. Between temperatures of 200 to 240 °C, the stress required for martensite transformation increases with temperature, and perfect superelasticity is observed due to the superelasticity. However, a partial recovery occurs at 260 °C since the martensite transformation and plastic deformation occurs simultaneously while the testing temperature is approaching M_d_. From 280 to 320 °C, the sample shows no recovery, but critical stress increases, suggesting that there is still phase transformation. Beyond 320 °C, ${\sigma }_{c}^{T}$ decreases and no recovery is observed due to the fact that plastic deformation of austenite takes place before martensite transformation, thus shape recovery cannot be found. The slope of the plateau region (due to phase transformation and/or plastic deformation), m_p_, is positive from 180 to 300 °C. At 320 °C, it is almost zero and then it is negative at higher temperatures. The critical stress decreases with temperature due to the conventional softening in metals at high temperature. Thus, the M_d_ temperature was determined to be around 300–320 °C, as it was the highest temperature that phase transformation was still observed. Due to the difficulty of determining the temperature where only plastic deformation takes place in stress-strain curves of SMAs, the intersection method is generally used. The M_d_ can determined to be 280 °C by this method.

Fig. 1b can be divided into five stages (I, II, III, IV, V) based on the deformation behavior. The transformation temperatures (TTs) obtained from DSC measurements are also shown in Fig. 1b, where the M_s_ is very close to the intersection (lowest) point between the line of low critical stress and stress induced phase transformation. From M_s_ to T_SE_, ${\sigma }_{c}^{T}$ increases linearly with temperature. From T_SE_ to M_d_, ${\sigma }_{c}^{T}$ increases and above M_d_, ${\sigma }_{c}^{T}$ decreases with temperature. The decrease of the stress can be explained by the increase in plasticity of austenite and dislocation mechanisms start to dominate.

### Indentation Load-Depth Curves

**Figure 2:**
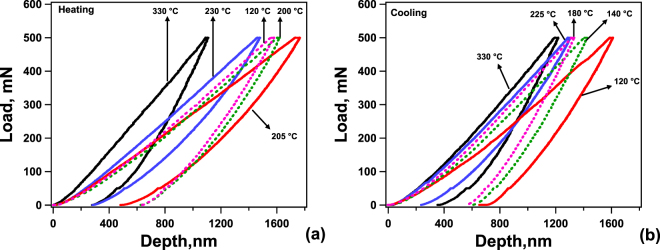
Spherical indentation responses of typical load-displacement relationships at selected temperature. Load-displacement curves of NiTiHf under load 500 mN with heating process from 28 °C to 340 °C (**a**) and cooling process from 340  to 28 °C (**b**).

Fig. 2 shows the load-displacement (L-D) curves for the spherical indentation response of the NiTiHf at temperatures between 30 and 340 °C. The data obtained at other temperatures are omitted in the figure for clarity. Fig. 2a and b are the L-D curves during heating and cooling processes, respectively. Most of the recent indentation studies with elevated temperatures only focused on the heating process^21,23,28,34,35^ to characterize the austenite start and finish temperatures of NiTi alloys and the cooling process has not been studied. Thus, a systematically and stepwise cooling process was followed after heating at 340 °C to identify the martensite phase transformation temperatures during cooling. At each temperature, indentations were repeated three times at different locations. For clarity, the first indentation load-displacement curve obtained at each temperature is displayed. The NiTiHf alloy starts in the pure martensitic phase in heating and austenite phase in cooling. It is instructive to note that these TTs extracted from the indentation response are not expected exactly match the TTs obtained from DSC as they would depend on the applied indentation force. Thus, the TTs obtained from indentation response will be named as ${M}_{s}^{I}$, ${M}_{f}^{I}$, ${A}_{s}^{I}$, and ${A}_{f}^{I}$ throughout the text.

In the beginning of the heating process as shown in Fig. 2a, the maximum indentation depth (D_max_) slightly increases with temperature from 120 to 200 °C, and then increases substantially at 205 °C due to the phase transformation. The increase of the maximum indentation depth indicates that the material is becoming softer which is consistent with the indentation hardness value displayed in Fig. 2 at each temperature. However, the hardness value does not change much with temperature, which might be due to the complex phases during phase transformation. Wood *et al*.^23^ also observed an increase in D_max_ below ${A}_{f}^{I}$. Above 205 °C, in Fig. 2a, austenite phase was formed completely and both the maximum depth of indentation and the remnant depth (D_R_) decreased, suggesting that the superelastic strain recovery mechanism was operating upon unloading only around ${A}_{f}^{I}$, but was absent at lower temperatures (below ${A}_{s}^{I}$). Meanwhile, the indentation hardness increased dramatically from 205 °C (2.8 GPa) to 230 °C (3.47 GPa), indicating austenite phase formation. Thus, we conclude that the 205 °C is the austenite finish temperature during indentation, which matches with the A_f_ (205 °C) measured from DSC of the bulk NiTiHf. The L-D curve at 230 °C provides the clearest evidence of the transition between superelastic and non-superelastic behavior, since it exhibits more depth recovery than the ones observed below 205 °C. With further increasing in temperature to 330 °C, the maximum indentation depth decreases dramatically, resulting in an increase in hardness due to dislocation-mediated plastic deformation.

**Figure 3:**
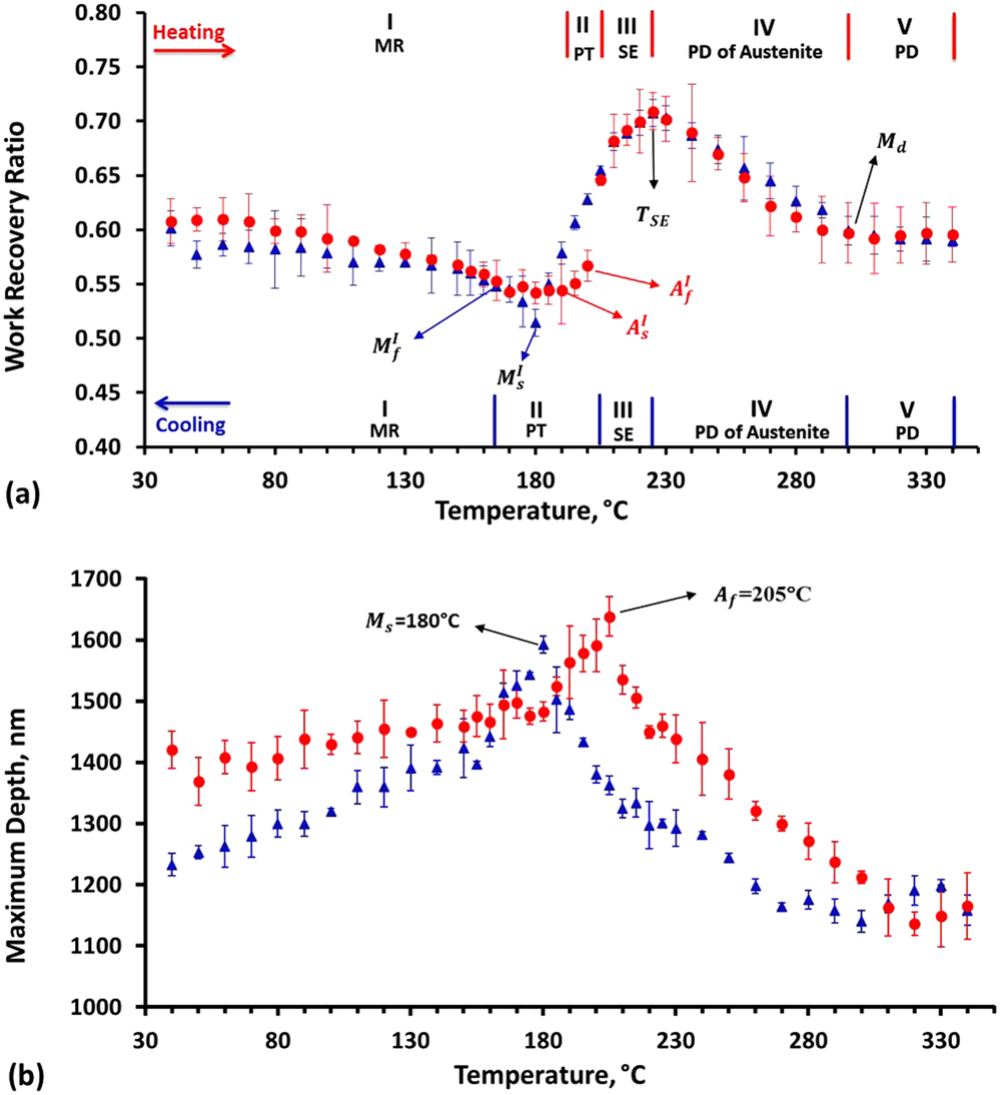
Spherical indentation response of work recovery ratio (**a**), maximum indentation depth D_max_ (**b**) as a function of temperature under 500 mN of NiTiHf with heating process from 28  to 340 °C marked as circle and cooling process from 340  to 30 °C marked as triangle.

Fig. 2b shows the L-D relation as a function of temperature during the cooling process. Starting at 330 °C, either the hardness or the maximum indentation depth is the same as that measured during the heating process. This may be caused by the variations in indentation locations or grain orientations. However, during the cooling process, the sample was in pure austenite initially and deformed plastically because the temperature was above M_d_. The maximum indentation depth increased slightly as the temperature decreased to 225 °C, where a small decrease in indentation hardness synchronously appeared even though temperature drops by 100 °C. A sudden increase in D_max_ and decrease in hardness happened when temperature decreased to 180 °C, where the lowest value of hardness and modulus were observed during the cooling process. Such a decrease also indicates the decrease in superelasticity. Furthermore, temperature below 180 °C shows an increase in D_max_ with decreasing temperature, corresponding to the decrease in hardness. Here, 180 °C is the ${M}_{s}^{I}$, during cooling, which is in good agreement with the ${M}_{s}^{I}$, (185 °C) measured from DSC. These predictions from the indentation L-D response of heating and cooling process will be discussed further in the next section.

## Discussion

To gain further understanding of the L-D curves, we evaluate indentation hardness, modulus, and recoverable ratio as a function of temperature. Indentation response of measured hardness and elastic moduli values are not the same as that of either martensite or austenite phases. Although indentation can cause phase transformation under the indenter, hardness and elastic moduli with a wide range of temperature cannot define the actual state of transforming phases or other stages (SE, plasticity, and martensite reorientation) accurately. However, the work/depth recovery or remnant depth recovery as a function of temperature are often used to characterize the transformation temperature and superelasticity^8,23^. In this article, the work recovery method was used to define the stages of mechanical deformation.

Fig. 3a and b are the temperature dependent spherical indentation response on work recovery and maximum indent depth (D_max_) of NiTiHf under peak load 500 mN, respectively. With a complete thermal cycling between 30 and 340 °C, indentation data was collected and the average work recovery ratio and maximum indent depth were analyzed from three repeated experiments. In order to interpret the data more accurate, error bars representing the standard deviation were shown at temperature. Based on the general behavior of SMAs under stress and temperature, the indentation work recoverable ratio of NiTiHf alloy as a function of temperature can also be separated into stages during heating and cooling, shown in Fig. 3a.

Starting with the heating process in pure martensite, martensite variant reorientation takes place during indentation with a peak load of 500 mN. The work recoverable ratio slightly decreases from 0.61 to 0.54 while the temperature increases from 30 to 180 °C (this temperature range was selected as stage I). In this temperature range, the maximum depth increased incrementally in Fig. 3b. An enormous increase (15%) of work recovery was observed from 190 to 225 °C. Such an increase in the work recovery ratio is due to the increased volume fraction of austenite phase in the matrix that results in SIM. As shown in Fig. 3a, the work recoverable ratio exhibits a sudden jump between 200 and 205 °C, manifesting the completion of austenite phase transformation at 200 °C. Therefore, the temperature ranges from 190 to 200 °C was selected as stage II (PT/VR), and 190 °C is the ${A}_{s}^{I}$, since it shows the lowest work recoverable ratio and it starts to increase at higher temperatures. It should be noted that maximum depth increased abruptly from 180 to 205 °C, as shown in Fig. 3b. It suggests that the volume fraction of the material that undergoes VR or SIM increased in this section. This observation can be attributed to the low critical stress requirement for SIM or VR around the transformation temperatures as shown in Fig. 1. Since the applied force is kept constant, lower critical stress resulted in larger volume fraction of transformation or variant reorientation. Above ${A}_{f}^{I}$, a dramatic increase in work recovery was observed, where the highest value indicates the best superelastic behavior upon unloading at 225 °C (T_SE_). Thus, the range 205–225 °C was defined as the superelastic region (stage III). Comparing with the superelastic behavior of the bulk sample, perfect superelasticity was found from 200–240 °C, as shown in Fig. 1a, which agrees well with the indentation results. It should be kept in mind that although the stress is constant in the compression test of the bulk sample, the stress is distributed in spherical indentation tests. Thus, in spherical indentation, a range of stress is applied to the material. It can also be argued that the end temperature of region III (T_SE_) will depend on the indenter size and the applied load. The higher load and lower indenter diameter will result in lower T_SE_. It was observed in another NiTiHf sample that when the applied load was increased from 500 mN to 2000 mN, T_SE_ was decreased by 25 °C (the results are not shown here). From Fig. 3b, it can be determined that D_max_ decreases above 205 °C and the intersection method can be used to find that 225 °C is the deflection point, above which the decrease in D_max_ is linear. Above 225 °C, the work recoverable ratio starts to decrease with temperature up to 300 °C, and then saturates to a constant work recoverable ratio of 0.61. Such a decrease is attributed to the increased plastic deformation of austenite phase with partial superelasticity, and it also indicates phase transformation. It should be noted that 300 °C was determined to be the M_d_ since there is no phase transformation at higher temperatures. Therefore, 225–300 °C was selected as stage IV (SIM + PD) and 300–340 °C is the stage V where only the plasticity will occur. D_max_ decreases linearly from 205 to 320 °C and then increases with temperature.

Following the heating experiments, the work recovery ratio as a function of temperature with cooling process was also divided into five stages as shown in Fig. 3a with triangle marks. During cooling from 340–225 °C, the indentation work recoverable ratio shows almost identical hysteresis with the heating experiments as the material is completely austenite. On the other hand, D_max_ during cooling is lower than the D_max_ during heating. The difference can be attributed to the fact that since indentation measures local properties, orientation of grains could affect the results. Heating and cooling experiments are conducted at two different regions of a polycrystalline sample, thus, D_max_ will depend on the orientation of the grains. Since work recoverable ratio is almost identical, this behavior indicates the deformation of austenite has a good agreement of heating and cooling experiments. Therefore, the stage V and IV of cooling process are in the same temperature range as with the heating process. Between 225–205 °C, the work recovery ratio decreases linearly while D_max_ increases with decreasing temperature and the results are identical to heating curve. This stage was selected as stage III (SE). The results between 205 to 180 °C are similar to the previous region, however, the changes are more profound than the results deviate from to heating curve. This behavior is expected since during cooling, initially, austenite is stable fully from ${A}_{f}^{I}$ to ${M}_{s}^{I}$ and partially from ${M}_{s}^{I}$ to ${M}_{f}^{I}$, while during heating, martensite is stable from ${M}_{f}^{I}$ to ${A}_{s}^{I}$ and then partially from ${A}_{s}^{I}$ to ${A}_{f}^{I}$. Thus, decreasing temperature in this region, increases the volume fraction of austenite phase that goes through SIM but decrease of martensite to austenite back transformation. At 180 °C, the lowest work recovery ratio and the maximum D_max_ were observed during cooling experiments, and it was selected as the ${M}_{s}^{I}$ where SMAs show the lowest critical stress. From 180–160 °C, the work recoverable ratio increases sharply and maximum depth decreases pronouncedly compared to the values below 160 °C. Such a temperature range was determined as stage II (PT). The martensite finish temperature ${M}_{f}^{I}$ was determined to be around 160 °C because the work recoverable ratio below that temperature (160 °C) was not increased much which means that the martensite phase transformation was fully completed. The smooth and steady curve below 160 °C shows a similar behavior as the heating process where martensite reorientation takes place, named as stage I. Comparing with the heating and cooling loops in Fig. 3a, the small hysteresis between 175 to 205 °C attributes to the thermal hysteresis of austenite and martensite.

It should be noted that shape memory and material properties are highly orientation dependent^31,36,37^. The average grain size of the tested material was around 50 µm, the indenter tip radius was 25 µm, the maximum contact diameter was around 20 µm and the distance between indent centers was 200 µm. Thus, it is possible that some indents will occurs inside the grains while others could hit the grain boundaries. Thus, indentation response at three varied locations gives different L-D results depending on the grain orientations. It is clear that the error bars in stage IV and V are relatively higher than other stages, especially in the heating process. Thus, it might be suggested that grain orientations are affecting the plastic deformation more than the shape memory response. There are only a few exceptions to this where the error bar at ${A}_{s}^{I}$ during heating is large since the indentation response will highly depend on whether, the material is austenite, martensite or mixed phase.

Nevertheless, the transformation temperatures of ${M}_{s}^{I}$, ${M}_{f}^{I}$, ${A}_{s}^{I}$, and ${A}_{f}^{I}$ from indentation response of work recovery can be determined as 180, 160, 190, and 200 °C, respectively. Moreover, T_SE_ can be determined as 225 °C but it should be noted that it will depend on the loading level as well as indentation tip radius. Lastly, M_d_ can be determined to be around 300 °C by using indentation technique. These values are in good agreement with the DSC and bulk mechanical compression results. However, it should be noted that, indentation technique can be used to determine local properties. Such a new method of characterizing shape memory properties has several advantages than the conventional method: i) the programming interface of indentation technique makes the experiments easier and save time for researchers, ii) it can be employed as nondestructive testing of bulk SMAs, iii) the small scale measurement economically saves material, iv) it can precisely determine local properties at nano or macro scales, v) the purging system avoids oxidization at high temperature.

### Summary

Spherical indentation technique was used to determine the temperature-dependent behavior of a high temperature NiTiHf shape memory alloy. This method can be used for detecting the transformation temperatures and deformation stages. The temperature, where shows the highest D_max_ during heating and cooling, corresponds to ${A}_{f}^{I}$ and ${M}_{s}^{I}$, respectively, while the temperature at the lowest work recoverable ratio during heating and cooling corresponds to ${A}_{s}^{I}$ and ${M}_{s}^{I}$, respectively. The TTs obtained through indentation are in very good agreement with the TTs obtained from DSC results. Moreover, the temperature with the highest work recoverable ratio is the T_SE_, and M_d_ can be determined at the temperature where the work recoverable ratio starts to saturate after T_SE_. Furthermore, the results obtained using the indentation method are in a good agreement with the phase transformation behavior performed on bulk materials under compression. This simple indentation technique is capable of quantitative characterization at micro- and nano-meter scales of shape memory materials.

## Method

The Ni_50.3_Ti_29.7_Hf_20_ alloy was inductively melted using a graphite crucible and cast into a 1 inch diameter copper chill mold. The ingot was homogenized at 1050 °C for 72 hours and extruded at 900 °C with a 7:1 reduction in area, and then aged at 550 °C for 3 hours. For simplicity, aged Ni_50.3_Ti_29.7_Hf_20_ is denoted as NiTiHf in this article. The alloys were electrical-discharge machined to a standard compression sample (4 mm × 4 mm × 8 mm). Mechanical testing was conducted in an MTS Landmark servo hydraulic test platform with a 100 kN load cell. Stress-free transformation temperatures were measured using a Perkin-Elmer Pyris 1 differential scanning calorimeter (DSC) with a scan rate of 10 °C min^−1^.

**Figure 4:**
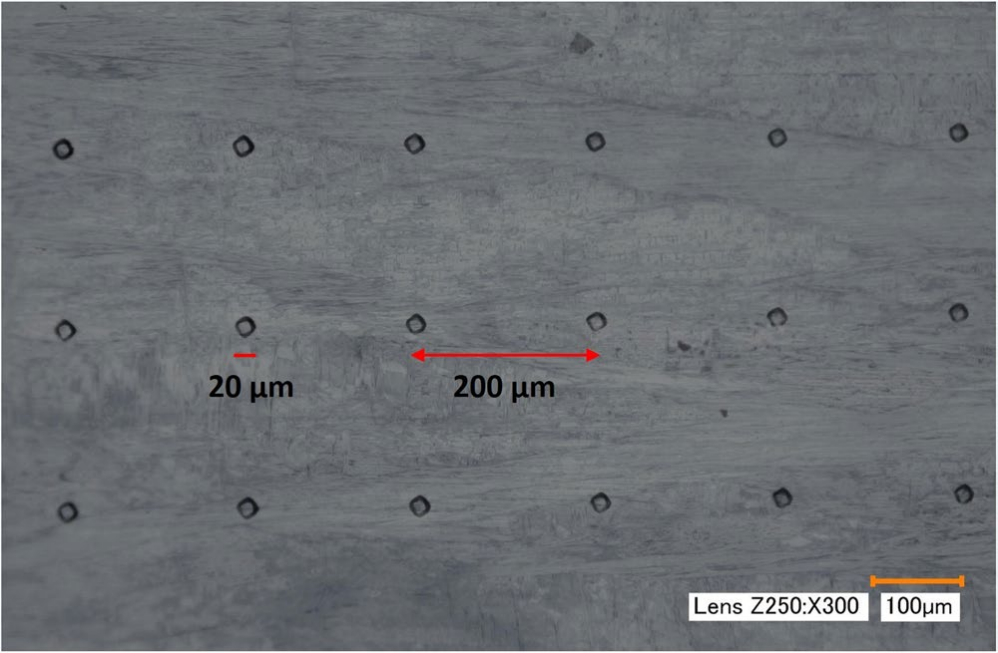
Optical microscopy image of aged Ni_50.3_Ti_29.7_Hf_20_ after indentation experiments.

Before indentation experiments, the surface roughness of samples was reduced to 0.05 µm in five steps by using Buehler EcoMet 250 Grinder-Polisher with an AutoMet 250 Power head. Spherical indents were made using the MicroMaterials Nanotest Platform equipped with a hot stage (up to 750 °C) for controlling the temperature of both indenter and sample. A tip radius of 25 µm diamond spherical indenter was mounted on a heating shield which reduces the heat transfer from indenter to the surrounding. The NiTiHf specimen was bounded to the hot stage using a thermally conductive ceramic paste. Thermocouples were mounted on the surface and directly above the resistive heating element to monitor and control specimen temperatures. To minimize transient thermal fluctuations and heat transfer between the specimen surface and indenter tip during experiments, a separate resistive heater thermal control system was used to heat the indenter. The temperature of the specimen surface and indenter was controlled to within 0.1 °C. The indentation platform was configured horizontally in order to minimize heat transfer. Prior to indentation, a standard load and depth were calibrated. When the temperatures of the indenter and the specimen were stabilized, indentation was made using a peak load of 500 mN with a loading rate of 5 mN/s. Following a dwelling period of 10 s at the peak load, the indenter was unloaded at the same rate of 5 mN/s. The spherical indenter and the specimen were heated simultaneously with a maximum heating rate of 8 °C/min. The temperature range of indentation experiments was from 30 to 340 °C with 10 °C intervals, while 5 °C intervals were used between 150 and 240 °C to obtain accurate phase transformation behavior. At each temperature, indentation experiments were repeated three times at the same load. Fig. 4 is the optical microscope image of the indentation location and indentation size. The average indent size is about 20 µm and the space between each indent is around 200 µm.

Hardness and elastic modulus were calculated from the initial stage of the unloading curve using the Nano Vantage Software^38^. The ratio of the recoverable energy to the total energy is defined as ${\eta }_{w}$. The recoverable energy, ${W}_{e}$, can be obtained by integrating the unloading curve. The total deformation energy, ${W}_{t}$, is the area under the loading curve. The area between the loading and unloading curve is the irrecoverable work, ${W}_{irr}$, also known as dissipation energy.
